# Serum inflammatory factors, vitamin D levels, and asthma severity in children with comorbid asthma and obesity/overweight: a comparative study

**DOI:** 10.3389/fped.2025.1439841

**Published:** 2025-02-27

**Authors:** Wan-yu Jiang, Rong-hong Jiao, Su-li Ma, Jin-sheng Dai, Hai-feng Zhu, Meng-ya Wu, Yan-ran Che, Lei Zhang, Xiao-yuan Ding

**Affiliations:** ^1^ Pediatrics Department, Shanghai Pudong New Area People’s Hospital, Shanghai, China; ^2^ Department of Clinical Laboratory, Shanghai Pudong New Area People’s Hospital, Shanghai, China

**Keywords:** vitamin D, obesity, asthma severity, pediatric asthma, inflammatory factors, retrospective comparative study

## Abstract

**Objective:**

To investigate serum inflammatory factors, vitamin D levels, and asthma severity in children with comorbid asthma and obesity/overweight, compared with those with asthma or obesity/overweight alone.

**Methods:**

This retrospective comparative study included children suffering from asthma alone, asthma combined with obesity/overweight, or obesity/overweight alone at Shanghai Pudong New Area People's Hospital between January 2020 and December 2021.

**Results:**

A total of 168 children (mean age: 4.32 ± 1.64 years; 117 males) were included. Compared with children with asthma alone (*n* = 56), those with comorbid asthma and obesity/overweight (*n* = 56) exhibited higher levels of serum levels of interleukin 6 (IL-6) (35.75 ± 24.56 vs. 15.40 ± 19.67), TNF-α (15.44 ± 7.35 vs. 12.16 ± 7.24), and leptin (3.89 ± 3.81 vs. 1.27 ± 2.31), and lower levels of 25-hydroxycholecalciferol (25-(OH) D_3_) (26.03 ± 10.77 vs. 37.15 ± 13.35), IL-10 (8.69 ± 2.76 vs. 15.32 ± 6.28), and IL-13 (449.40 ± 315.37 vs. 605.27 ± 351.02) (all *P* < 0.05). Compared with children with obese/overweight alone (*n* = 56), those with comorbid asthma and obesity/overweight had lower IL-10 (8.69 ± 2.76 vs. 12.29 ± 6.61) and higher IL-6 (35.75 ± 24.56 vs. 20.53 ± 17.07), IL-13 (449.40 ± 315.37 vs. 309.47 ± 257.45), and leptin (3.89 ± 3.81 vs. 2.48 ± 3.52) (all *P* < 0.05). Children with comorbid asthma and obesity/overweight showed higher Preschool Respiratory Assessment Measure (PRAM) scores (3.14 ± 2.40 vs. 1.93 ± 1.02, *P* = 0.008) and longer hospital stays (5.96 ± 1.25 vs. 5.29 ± 1.36 days, *P* = 0.007) compared to those with asthma alone.

**Conclusions:**

Significant differences were observed in IL-6, IL-10, IL-13, 25-(OH) D_3_ levels, and leptin among children with asthma combined with obesity/overweight and those with asthma or obesity/overweight alone. Children with obesity/overweight alone displayed more severe clinical manifestations and longer hospital stays compared with those with asthma alone.

## Introduction

Pediatric asthma is a chronic inflammatory disease of the airways in children, characterized by airflow obstruction. According to the Global Asthma Report (GAR), 262 million people had asthma in 2019, and 1 in 10 children had asthma symptoms worldwide (1). The global epidemic of asthma in children and adults continues to rise (2). Environmental factors (such as air pollution, pollens, mold, pets, and weather conditions), host factors (such as obesity, nutritional factors, infections, and allergic sensitization), and genetic factors (i.e., asthma susceptibility genes) interact to influence the occurrence and severity of asthma (3).

Childhood obesity is another serious public health issue worldwide, putting children and adolescents at risk of poor health during childhood and adulthood. In China, 6.8% of children <6 years are overweight and 3.6% are obese. In the recent decade, the prevalence of overweight and obesity among Chinese children has been increasing (4). Among children and adolescents aged 6–17 years, 11.1% are overweight and 7.9% are obese (5). Obesity/overweight is a major risk factor for asthma and a complex interaction between obesity and asthma results in higher severity of asthma and poorer control of asthma symptoms (6). In obese individuals, multiple inflammatory mediators and M1 macrophage infiltration are increased in adipose tissues, causing inflammation. The inflammatory process also increases the synthesis of proinflammatory cytokines such as interleukin (IL)-6, tumor necrosis factor-α (TNF-α), IL-1β, and transforming growth factor-β (TGF-β), all contributing to asthma pathogenesis and severity (7). Obesity-related asthma is associated with a Th1 immune response (involving TNF-α, IFN-γ, IL-6, and IL-8) rather than a Th2 response (which involved IL-4, IL-5, IL-10, and IL-13) (8). Nevertheless, the role of inflammatory factors in obese patients with asthma remains unclear.

Vitamin D is an essential nutrient and is required for immune regulation (9, 10). 25-hydroxycholecalciferol (25-(OH) D_3_) is a form of vitamin D that the body produces or absorbs from animal sources. The other form of vitamin D is 25-hydroxyvitamin D_2_ (ergocalciferol), which comes from plant sources. The total level of 25-(OH)D_3_ in the blood is the primary measurement used to assess vitamin D status (11). Obesity and vitamin D deficiency (serum levels <30 ng/ml) have been associated with more severe asthma symptoms (12, 13). The Childhood Asthma Management Program (CAMP) revealed that 35% of children aged 5–12 years had mild to moderate asthma and vitamin D deficiency (<30 ng/ml) (14). Moreover, a study that used the National Health and Nutrition Examination Survey (NHANES) found a significant correlation between vitamin D deficiency [defined as serum 25-(OH) D_3_ levels <30 ng/ml] and asthma symptoms in children. Another study associated vitamin D deficiency with poor lung function in obese children (15). Taken together, vitamin D deficiency is seen in obesity and asthma, but the exact interplay among the three in children remains to be defined.

Therefore, this study aimed to investigate serum inflammatory factors, vitamin D levels, and asthma severity in children with comorbid asthma and obesity/overweight, compared with those with asthma or obesity/overweight alone. The results could help refine our understanding of the epidemiology and pathogenesis of asthma in children with obesity/overweight.

## Methods

### Study design and patients

The retrospective comparative study included children with asthma alone, comorbid asthma and obesity/overweight, or obesity/overweight alone, hospitalized (those with asthma) or attending the outpatient clinic (those with obesity/overweight alone) at Shanghai Pudong New Area People's Hospital between January 2020 and December 2021. This study was approved by the Ethics Committee of Shanghai Pudong New Area People's Hospital (approval #prylz2020-101). The requirement for individual consent was waived by the committee due to the retrospective nature of the study.

The inclusion criteria were (1) children aged 1–11 years and (2) diagnosed with asthma according to the criteria in the “Guideline for the Diagnosis and Optimal Management of Asthma in Children (2016)” (16). The exclusion criterion was wheezing due to other conditions (e.g., congenital heart disease, gastroesophageal reflux, or bronchopulmonary dysplasia).

### Data collection

Obese/overweight was defined according to the “Chinese Preschool Children Growth Reference Standard and Related Curve: Based on GAMLSS Approach” (17). A BMI of ≥P_95_ was considered obese, and a BMI of ≥P_85_ was considered overweight (17). The children were categorized into children with asthma alone, obese/overweight alone, and obese/overweight and asthma comorbidity (hereafter referred to as those with comorbid asthma and obesity/overweight). The age, sex, height, and weight of the selected subjects were recorded. Age was accurate to 1 month, height to 1 cm, and weight to 0.1 kg. The PRAM score, length of hospital stay, hospitalization cost, and systemic glucocorticoid use were collected in children with asthma. The discharge criteria included no symptoms of wheezing or shortness of breath and the absence of pulmonary rales. The Preschool Respiratory Assessment Measure (PRAM) was used to evaluate asthma severity (18).

On the day of hospital admission, blood was collected for laboratory testing. Fasting venous blood (3 ml) was collected, held at room temperature for 30 min, and centrifuged at 3,000 r/min for 5 min to collect the serum. Separated serum was stored at −80℃ until further use. The 25-(OH) D_3_ levels were determined using the electrochemical luminescence method from Roche (Basel, Switzerland). Leptin (kit no. CSB-E04649h), IL-6 (kit no. CSB-E04638h), TNF-α (kit no. CSB-E04740h), IL-10 (kit no. CSB-E04593h), IL-4 (kit no. CSB-E04633h), and IL-13 (kit no. CSB-E04601h) levels were determined by ELISA, following manufacturer's instructions (Cusabio Technology LLC, Wuhan, China). A Microlab STAR automatic enzyme immunoassay analyzer (Hamilton Co., Reno, NV, USA) was used for measurements.

### Sample size

The sample size for this study was calculated to ensure sufficient power to detect significant differences in serum inflammatory factors, vitamin D levels, and asthma severity among the three groups: asthma alone, asthma combined with obesity/overweight, and obesity/overweight alone.

According to preliminary investigation data by the authors (10 cases in each group), the mean levels of IL-6 were 13.45 ± 17.69 pg/ml for asthma, 39.67 ± 21.58 pg/ml for asthma and obesity, and 22.05 ± 22.13 pg/ml for obesity. Using the G*Power 3.1.9.2 software, the effect size (Cohen's d for ANOVA) was calculated as 0.49. Using a two-sided significance level (α) of 0.05 and a power (1-β) of 0.90, it was calculated that a total of 57 patients would be needed. Considering that IL-6 levels do not conform to a normal distribution, the number of patients needed to be increased by 15%. In addition, considering a missing rate of 10%, the minimum sample size required was *n* = 57 × 115% × 110%, which is approximately equal to 24 patients in each group. Based on the financial and material resources of this study, 56 samples were surveyed in each group to meet the minimum sample size requirement.

### Statistical analysis

SPSS 23.0 (IBM, Armonk, NY, USA) was used for statistical analysis. The figures were drawn using the “ggplot2” 3.4.4 package in R 4.3.1. Continuous variables were described as means ± SD. Normally distributed variables were tested using the independent sample t-test (two groups) and one-way ANOVA (multiple groups). For data with a non-normal distribution, the Mann–Whitney *U* test was used to compare two groups, and the Kruskal–Wallis H test was used to compare multiple groups. The Bonferroni method was used to control for false positives. Categorical variables were described as *n* (%) and analyzed using the chi-squared test or Fisher's exact test. Fisher's exact test when the expected number of observations in any of the cells of a contingency table was below 5, or below 10 when there was only one degree of freedom; otherwise, the chi-squared test was used. Therefore, the chi-squared test was used for sex and systemic glucocorticoid use. Spearman correlation analysis was conducted to analyze the correlations among variables. “Systemic glucocorticoid use” is a categorical (binary) variable; after it was encoded as 0/1, the Spearman correlation coefficient with vitamin D was calculated, which is a point-biserial correlation coefficient. Two-sided *P*-values < 0.05 were considered statistically significant.

## Results

### Characteristics of the children

The eligible patients treated between January 2020 and December 2021 were included. A total of 168 children (117 males; mean age of 4.32 ± 1.64 years) were included. The patients were grouped according to their condition: asthma, obese/overweight, and comorbid asthma and obese/overweight. There were no significant differences in sex or age among the three groups (all *P* > 0.05). Compared with children with asthma alone (*n* = 56), those with comorbid asthma and obesity/overweight (*n* = 56) and obesity/overweight (*n* = 56) had significantly higher BMI (19.70 ± 2.33 and 19.35 ± 2.20 vs. 16.16 ± 0.71, both *P* < 0.05) (Table 1).

**Table 1:** Demographic characteristics.

Variables	Asthma group (*n* = 56)	Comorbidity group (*n* = 56)	Obese/overweight group (*n* = 56)	*P*
Age (y)	4.39 ± 1.57	4.46 ± 1.77	4.13 ± 1.59	0.262
Sex				0.554^a^
Male	40 (71.4%)	36 (64.3%)	41 (73.2%)	
Female	16 (28.6%)	20 (35.7%)	15 (26.8%)	
Body mass index (kg/m^2^)	16.16 ± 0.71	19.70 ± 2.33*	19.35 ± 2.20*	<0.001

The *p*-values presented in the final column of the table were unadjusted. The *p*-values denoted with *signified *post-hoc* comparisons that were adjusted through the Bonferroni correction method.

* *P* < 0.05 vs. children with asthma alone.

^a^ Chi-squared test.

### Serum cytokine levels

**Figure 1:**
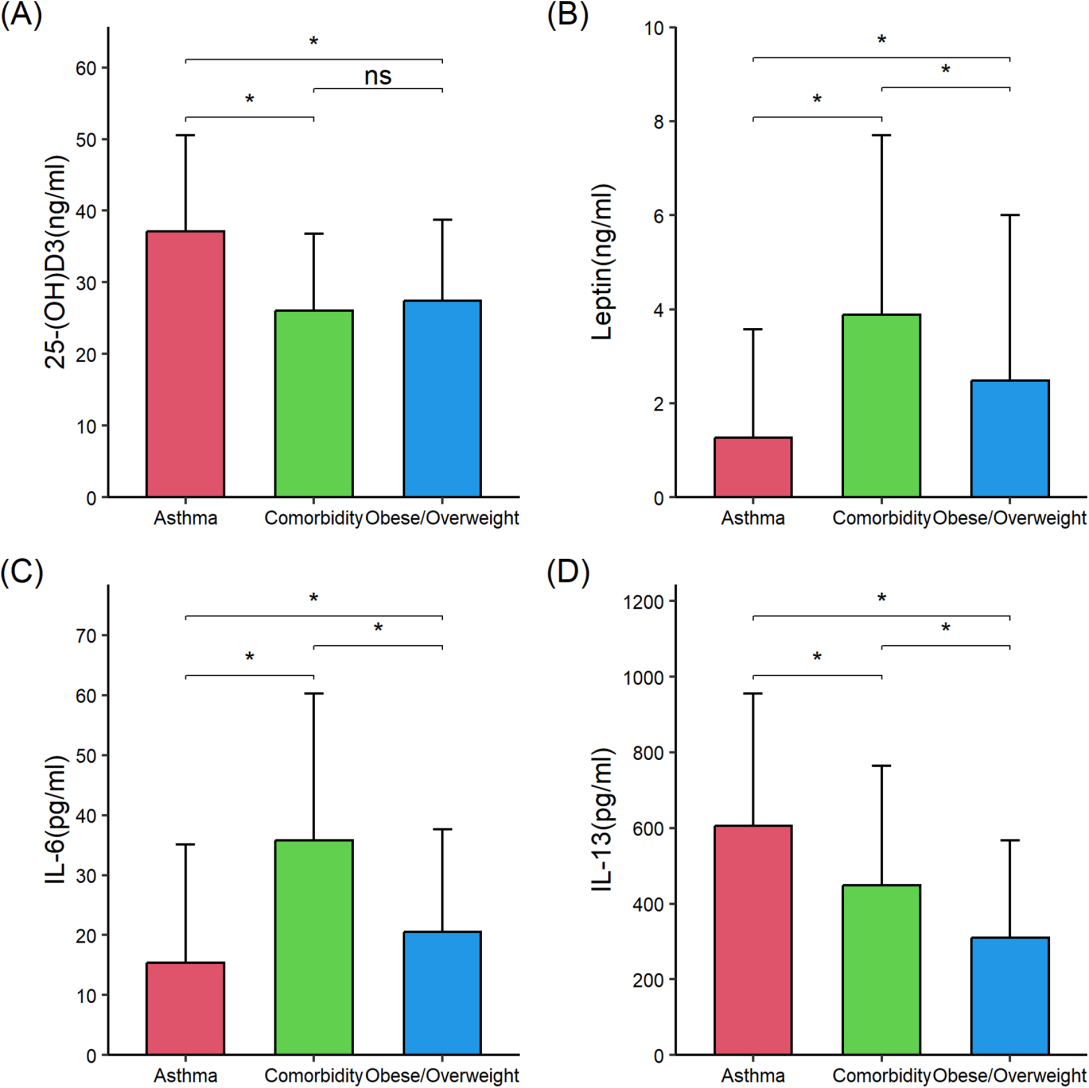
Comparison of 25-(OH) D3, leptin, interleukin (IL)-6, and IL-13 in the three groups of children. **(A)** 25-(OH)D_3_. **(B)** Leptin. **(C)** IL-6. **(D)** IL-13. 25-(OH) D_3_, 25-hydroxycholecalciferol; IL-6, interleukin 6; IL-13, interleukin 13. The data were non-normally distributed. The global *P*-values were from the Kruskal–Wallis test. The Mann–Whitney test was used for the *post hoc* testing of pairs of groups. Statistical significance between groups is indicated as follows: *P < 0.05; ns (not significant) *P* ≥ 0.05. The figure was drawn using the “ggplot2” 3.4.4 package in R 4.3.1. Error bars represented standard deviation.

**Figure 2:**
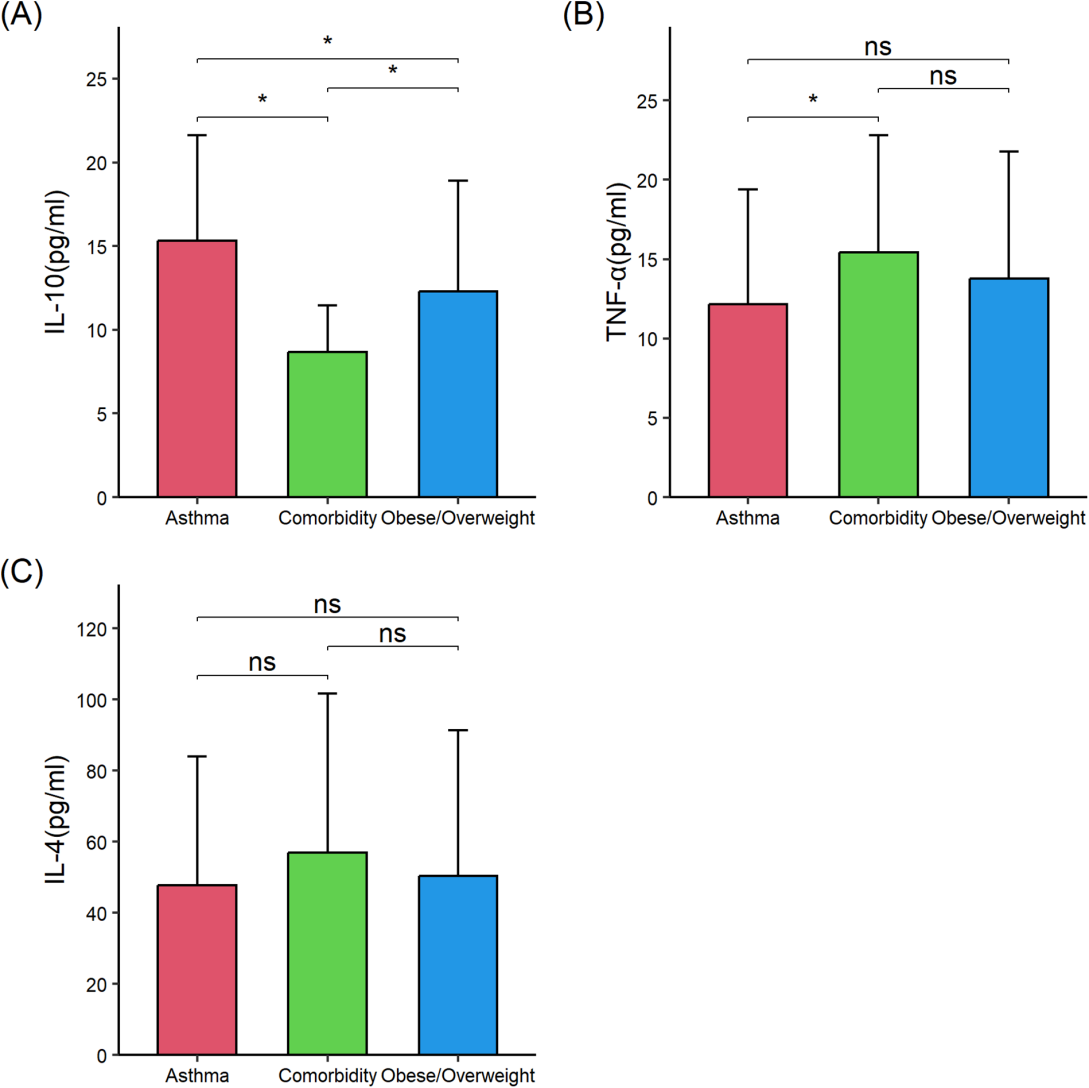
Comparison of interleukin (IL)-10, tumor necrosis factor (TNF)-*α*, and IL-4 in the three groups of children. **(A)** IL-10. **(B)** TNF-α. **(C)** IL-4. IL-10, interleukin 10; TNF-α, tumor necrosis factor-α; IL-4, interleukin 4. The data were non-normally distributed. The global *P*-values were from the Kruskal–Wallis test. The Mann–Whitney test was used for the *post hoc* testing of pairs of groups. Statistical significance between groups is indicated as follows: * *P* < 0.05; ns (not significant) *P* ≥ 0.05. The figure was drawn using the “ggplot2” 3.4.4 package in R 4.3.1. Error bars represented standard deviation.

Then, serum levels of proinflammatory and anti-inflammatory cytokines were compared among the three groups to determine whether comorbid asthma and obesity/overweight influenced the cytokine levels compared with asthma alone or obesity/overweight alone. Compared with children with asthma alone (*n* = 56), those with comorbid asthma and obesity/overweight (*n* = 56) exhibited significantly higher levels of serum levels of interleukin 6 (IL-6) (35.75 ± 24.56 vs. 15.40 ± 19.67), TNF-α (15.44 ± 7.35 vs. 12.16 ± 7.24), and leptin (3.89 ± 3.81 vs. 1.27 ± 2.31), and lower levels of 25-(OH) D_3_ (26.03 ± 10.77 vs. 37.15 ± 13.35), IL-10 (8.69 ± 2.76 vs. 15.32 ± 6.28), and IL-13 (449.40 ± 315.37 vs. 605.27 ± 351.02) (all *P* < 0.05). Compared with children with obese/overweight alone (*n* = 56), those with comorbid asthma and obesity/overweight had significantly lower IL-10 (8.69 ± 2.76 vs. 12.29 ± 6.61) and higher IL-6 (35.75 ± 24.56 vs. 20.53 ± 17.07), IL-13 (449.40 ± 315.37 vs. 309.47 ± 257.45), and leptin (3.89 ± 3.81 vs. 2.48 ± 3.52) (all *P* < 0.05) (Table 2; Figures 1, 2). These results suggested the combined effect of comorbid asthma and obesity/overweight on the levels of several cytokines.

**Table 2:** Comparison of proinflammatory mediators and 25-(OH) D3 in the three groups of children.

Indicators	Asthma group (*n* = 56)	Comorbidity group (*n* = 56)	Obese/overweight group (*n* = 56)	*P*
25-(OH) D_3_, ng/ml	37.15 ± 13.35	26.03 ± 10.77*	27.45 ± 11.27*	<0.001
IL-6, pg/ml	15.40 ± 19.67	35.75 ± 24.56*	20.53 ± 17.07*^,^#	<0.001
TNF-α, pg/ml	12.16 ± 7.24	15.44 ± 7.35*	13.77 ± 8.03	0.013
IL-10, pg/ml	15.32 ± 6.28	8.69 ± 2.76*	12.29 ± 6.61*^,^#	<0.001
IL-13, pg/ml	605.27 ± 351.02	449.40 ± 315.37*	309.47 ± 257.45*^,^#	<0.001
Leptin, ng/ml	1.27 ± 2.31	3.89 ± 3.81*	2.48 ± 3.52*^,^#	<0.001
IL-4, pg/ml	47.80 ± 36.15	56.90 ± 44.83	50.33 ± 40.93	0.741

25-(OH) D_3_, 25-hydroxycholecalciferol; IL-6, interleukin 6; TNF-α, tumor necrosis factor-α; IL-10, interleukin 10; IL-13, interleukin 13; IL-4, interleukin 4.

The *p*-values presented in the final column of the table were unadjusted. The *p*-values denoted with * and # signified *post-hoc* comparisons that were adjusted through the Bonferroni correction method.

The data were non-normally distributed. The global *P*-values were from the Kruskal–Wallis test. The Mann–Whitney test was used for the *post hoc* testing of pairs of groups: *P < 0.05 vs. the asthma group; ^#^*P* < 0.05 vs. the comorbidity group.

### Asthma symptoms and severity

In order to investigate whether obesity/overweight influenced asthma, the severity of asthma was compared between the asthma alone and comorbid asthma and obesity/overweight groups to examine the impact of obesity/overweight on asthma. Compared with children with asthma alone, those with comorbid asthma and obesity/overweight had significantly higher PRAM scores (3.14 ± 2.40 vs. 1.93 ± 1.02, *P* = 0.008) and longer length of hospital stay (5.96 ± 1.25 vs. 5.29 ± 1.36, *P* = 0.007). There were no significant differences in hospitalization cost and systemic glucocorticoid use between children with asthma alone and comorbid asthma and obesity/overweight (Table 3). Hence, obesity/overweight appeared to exacerbate asthma.

**Table 3:** Comparison of asthma severity and other parameters between children with asthma alone and those with comorbid asthma and obesity/overweight.

Parameters	Asthma group (*n* = 56)	Comorbidity group (*n* = 56)	*P*
PRAM score	1.93 ± 1.02	3.14 ± 2.40	0.008^a^
Length of hospital stay (d)	5.29 ± 1.36	5.96 ± 1.25	0.007^a^
Hospitalization cost (CNY)	3,593.79 ± 772.62	3,656.74 ± 720.22	0.364^a^
Systemic Glucocorticoid use	27 (48.2%)	35 (62.5%)	0.128^b^

PRAM, the preschool respiratory assessment measure.

^a^ Mann–Whitney test.

^b^ Chi-squared test.

### Correlations

Finally, correlations were examined between indicators of obesity and inflammation markers. BMI was negatively correlated with 25-(OH) D_3_ (*r* = −0.284, *P* < 0.001), IL-10 (*r* = −0.181, *P* = 0.019), and IL-13 (*r* = −0.188, *P* = 0.015). BMI was positively correlated with IL-6 (*r* = 0.386, *P* < 0.001), TNF-α (*r* = 0.172, *P* = 0.026), and leptin (*r* = 0.383, *P* < 0.001) (Table 4). Vitamin D was negatively correlated with IL-6 (*r* = −0.160, *P* = 0.038) and leptin (*r* = −0.155, *P* = 0.045). Vitamin D was positively correlated with IL-10 (*r* = 0.229, *P* = 0.003) (Table 5).

**Table 4:** Correlation of BMI with vitamin D and proinflammatory mediators.

	Correlation coefficient (r)	*P*
25-(OH) D_3_	−0.284	<0.001
IL-6	0.386	<0.001
TNF-α	0.172	0.026
IL-10	−0.181	0.019
IL-13	−0.188	0.015
Leptin	0.383	<0.001

25-(OH) D_3_, 25-hydroxycholecalciferol; IL-6, interleukin 6; TNF-α, tumor necrosis factor-α; IL-10, interleukin 10; IL-13, interleukin 13.

**Table 5:** Correlation of vitamin D with pro inflammatory mediators and clinical characteristics.

	Correlation coefficient (r)	*P*
IL-6	−0.160	0.038
TNF-α	−0.012	0.875
IL-10	0.229	0.003
IL-13	0.126	0.104
Leptin	−0.155	0.045
PRAM score	0.089	0.353
Length of hospital stay (d)	0.076	0.424
Hospitalization cost (CNY)	−0.091	0.343

IL-6, interleukin 6; TNF-α, tumor necrosis factor-α; IL-10, interleukin 10; IL-13, interleukin 13.

## Discussion

The present study showed that children with comorbid asthma and obesity/overweight had higher IL-6, TNF-α, and leptin, and lower 25-(OH) D_3_, IL-10, and IL-13 than children with asthma alone, and had lower IL-10 and higher IL-6, IL-13, and leptin than children with obesity/overweight alone. Compared with children with asthma alone, those with comorbid asthma and obesity/overweight had a higher PRAM score and longer length of hospital stay. These findings highlight the importance of obesity in the management of pediatric asthma.

In the current study, children with obesity/overweight and children with comorbid obesity/overweight and asthma showed higher IL-6 than children with asthma, which illustrates that IL-6 is involved in the pathogenesis of asthma in obese children. High IL-6 secretion by brown fat cells in mice causes the failure of brown fat cells to decompose fat or metabolize glucose and other substances, resulting in obesity and other related complications (19), supporting the present study. In addition, previous studies showed that obese children had a higher Th1 proportion and inflammatory cytokines (IL-6, IFN-γ, and TNF-α) levels (20, 21).

In this present study, the leptin levels in those with comorbid asthma and obesity/overweight were substantially higher than in children with obesity/overweight alone, while leptin expression in children with obesity/overweight alone was higher than in children with asthma alone. The comorbid asthma and overweight/obesity group had a lower expression of IL-10 and IL-13 than children with asthma alone, suggesting that the expression of Th2-related inflammatory factors was higher in children with asthma but not in children with comorbidity. However, the present study did not include healthy controls to verify the mechanism of leptin involvement in asthma. As observed in the literature, obese individuals have higher leptin expression, which in turn stimulates fat cells to secrete proinflammatory mediators such as IL-6, TNF-α, and IL-12 (22, 23). Leptin stimulates the secretion of IL-6 and TNF-α in human peripheral blood mononuclear cells (24). Leptin levels are higher in patients with asthma than in healthy controls, and leptin expression is significantly higher in patients with worsening asthma symptoms than in asymptomatic patients (25). Taken together, these findings suggest that leptin is involved in the pathogenesis of obesity-related asthma. Leptin promotes the differentiation and activation of Th1 cells, inhibits the production of the Th2 cytokines (such as IL-4, IL-5, and IL-10), and activates the proinflammatory Th17 cytokines (26–28).

In the present study, BMI was moderately and positively correlated with leptin and IL-6. A previous study found that the obesity-related proinflammatory cytokine IL-6 was related to asthma severity when metabolic syndrome co-occurred (29). Vitamin D reduces inflammation through various mechanisms, including inhibition of NF-κB signaling, P38 MAP kinase phosphorylation, and activating macrophages, B cells, T cells, neutrophils, dendritic cells, and mast cells (30, 31). Moreover, vitamin D deficiency was found to be associated with obesity and asthma (14). Vitamin D increases the expression of CD14 in lung epithelial cells and macrophages and participates in local defense mechanisms (32). Vitamin D regulates the expression of bronchial vascular endothelial growth factor, fibronectin, and IL-6 in bronchial smooth muscle, thereby reducing airway inflammation (33–35). Children with asthma and obesity have been reported to have lower serum vitamin D levels (15, 36). In a previous study, 29% of overweight children (BMI: 85th–95th percentile for age and gender), 34% of obese children (BMI: 95th–99th percentile), and 49% of severely obese children (BMI > 99th percentile) had 25(OH) D_3_ levels of <30 ng/ml (36). Serum vitamin D levels are inversely correlated with body fat levels, partly due to increased vitamin D storage in adipose tissue (37). Vitamin D deficiency is associated with the acute exacerbation of asthma and glucocorticoid resistance (38, 39). Vitamin D increases the bioavailability of glucocorticoids in airway smooth muscle cells and exerts a protective effect on Th1/Th2-driven airway inflammation (40). Nevertheless, the interaction between asthma, obesity, and vitamin D deficiency is complex.

In the present study, BMI was negatively correlated with 25-(OH) D_3,_ while the levels of 25-(OH) D_3_ in children with comorbid asthma and obesity/overweight and children with obese/overweight alone were lower than children with asthma alone and the normal standard (<30 ng/ml). Nevertheless, there were no significant differences between those with comorbid asthma and obesity/overweight and children with obesity/overweight alone. The present study examined the correlation between vitamin D and various proinflammatory factors, and vitamin D had a weak negative correlation with IL-6 and leptin, as well as a weak positive correlation with IL-10. Krajewska et al. found that vitamin D intake seemed to exert its anti-inflammatory effect mainly via decreasing the CRP level and protecting stable values of IL-10 (41). It suggests that 25-(OH)D_3_ may be involved in the regulation of relevant inflammatory factors. Still, vitamin D levels were not associated with the severity or length of hospital stay in children with asthma. Overall, vitamin D deficiency hinders the immune regulation in obese children.

According to the PRAM scores, the children with comorbidity suffered from more severe asthma symptoms, including difficulty breathing, compared with children with asthma alone. Nevertheless, systemic glucocorticoid administration did not produce any significant difference in asthma severity between the two groups. Children with comorbidity stayed longer in the hospital and suffered from more severe asthma symptoms with no ease with glucocorticoid administration, implying that obesity-related asthma may have had glucocorticoid resistance. Obese children displayed more severe clinical manifestations, and asthma control was very difficult in those children (42). The interaction between obesity and pulmonary disorders is multifaceted. Obesity alters chest wall dynamics, directly affecting the thorax biomechanics. In a previous study, obesity was positively associated with the length of hospital stay and the need for mechanical ventilation in children with asthma (43).

The present study has some limitations. Due to the absence of a normal control group in this study, it was impossible to compare the inflammatory factors of the disease groups with children without asthma or obesity/overweight. Since the data were retrieved from a single center, generalizability is limited. The study did not investigate the physical activity of the included children, family economy, or culture. Since it was a retrospective study, the families could not be contacted to collect such data. This study did not reexamine the inflammatory factors during the stable phase of asthma; therefore, the changes in the expression of these inflammatory factors after the treatment could not be speculated. Further prospective studies are necessary to investigate whether vitamin D supplementation or effective control of body weight can normalize the inflammatory factors and ease the severity of asthma in children with obesity-associated asthma.

In conclusion, there were significant differences in IL-6, IL-10, IL-13, 25-(OH) D_3_ levels, and leptin levels among children with asthma combined with obesity/overweight and those with asthma or obesity/overweight alone. Furthermore, those with obesity/overweight and asthma may display more severe clinical manifestations and longer hospital stays compared to children with asthma alone.

## Data Availability

The original contributions presented in the study are included in the article/Supplementary Material, further inquiries can be directed to the corresponding author.
